# Ignorability for general longitudinal data

**DOI:** 10.1093/biomet/asx020

**Published:** 2017-05-08

**Authors:** D. M. Farewell, C. Huang, V. Didelez

**Affiliations:** Division of Population Medicine, School of Medicine, Cardiff University, Heath Park, Cardiff CF14 4YS, U.K; Centre for Trials Research, College of Biomedical and Life Sciences, Cardiff University, Heath Park, Cardiff CF14 4YS, U.K; Leibniz Institute for Prevention Research and Epidemiology – BIPS, Achterstraße 30, 28359 Bremen, Germany

**Keywords:** Ignorability, Longitudinal data, Missing at random

## Abstract

Likelihood factors that can be disregarded for inference are termed ignorable. We demonstrate that close ties exist between ignorability and identification of causal effects by covariate adjustment. A graphical condition, stability, plays a role analogous to that of missingness at random, but is applicable to general longitudinal data. Our formulation of ignorability does not depend on any notion of missing data, so is appealing in situations where missing data may not actually exist. Several examples illustrate how stability may be assessed.

## 1 Introduction

We consider the analysis of longitudinal data. For each of a set of subjects, a sequence of observations is recorded, corresponding to the same property or feature of the subject evaluated at different times. Longitudinal data are common in scientific investigations, and their analysis has received much attention (Lindsey, 1999; Diggle et al., 2002; Molenberghs & Verbeke, 2006).

Longitudinal studies typically propose a schedule of measurement times in advance. Nevertheless, even in carefully designed experiments, the precise number and timings of observations are rarely completely determined by the investigator. Most obviously, a subject who dies during the course of a study can give rise to no further observations, scheduled or otherwise. In panel studies, a subject may fail to present for evaluation at an intermediate wave, but then return for final assessment at a later date. More generally, measurements may be recorded on quite arbitrary occasions and determined by convenience to the particular subject, availability of the investigator, or external factors such as weather conditions and public holidays.

In addition to external circumstances, observation times may be influenced by internal, subject-specific factors. Mood, medical intervention or indeed the biological processes underlying the longitudinal assessments can all affect the likelihood of recording an observation. An important special case is informative drop-out, where attrition relates to health (Sun et al., 2012).

Dependence between timings and observations may still be ignorable, in the sense that certain likelihood-based analyses are equivalent under such dependence. Ignorability is ordinarily described within a missing data framework, which presupposes that the missing data actually exist (Little & Rubin, 2002, p. 8). Especially for longitudinal data, this is only very rarely the case; more usually, missing data constitute a convenient and sometimes compelling fiction.

We argue that this fiction is not needed. Instead, we provide an alternative characterization of ignorability for general longitudinal data that does not depend on any notion of missing data. We do so by applying the machinery of causal inference (Pearl, 2009; Dawid & Didelez, 2010) to the components of a marked point process (Jacobsen, 2006). We caution that suppression of the usual missing data machinery does not absolve the analyst of attention to closely related matters, the challenges of which are perhaps even more starkly obvious within our causal formulation.

For concreteness, consider the longitudinal measurement of foetal size. In early pregnancy, foetal growth is often monitored using the crown-rump length, assessed electronically from an ultrasound image. Crown-rump length could in principle be measured at any point after conception. We stress, however, that crown-rump length does not actually exist at every point after conception. This is most obvious following birth, when it is no longer meaningful to measure crown-rump length by ultrasound. However, our argument is more general: no unambiguous, unique definition of crown-rump length can be made except on those occasions when it is actually measured. Certainly, a healthy foetus has a shape and size that is complex and growing more or less continuously, but crown-rump length is not simply a one-dimensional slice of this high-dimensional, continuous-time process; it is an external procedure subject to many influences apart from foetal size, including the skill of the sonographer, the resolution of the ultrasound, and the cooperation of the foetus. Consequently, crown-rump length only exists in a meaningful way on precisely those occasions when it is measured. To fix ideas, we refer to this example throughout.

## 2 Notation

We omit subject-specific subscripts *i*, and let (*t*, *y*) be a marked point process (Jacobsen, 2006, p. 10) representing the longitudinal data arising from a particular subject. The increasing sequence *t* = (*t*_1_, *t*_2_, …) is a standard point process, and records the observation times for this subject. The sequence *y* = (*y*_1_, *y*_2_, …) contains the corresponding longitudinal measurements. We resist the temptation to define for each of the *n* subjects an underlying continuous or complete measurement process; the existence or otherwise of this complete measurement process, however it might be defined, is irrelevant in our subsequent development.

Following Jacobsen (2006), we allow only finitely many longitudinal measurements in any finite time interval, and define *t_j_* = ∞ if fewer than *j* events occur altogether; drop-outs require no special handling. If *t_j_* = ∞, we assign to the corresponding *y_j_* the irrelevant mark ∇. Defining *m* = max{*j* : *t_j_* < ∞}, we formally admit the possibility that *m* = 0 and no measurements are made on a particular subject, but this case is usually of little interest. In our crown-rump length example, an idealized realization (*t*, *y*) of the marked point process in which *m* = 2 might comprise the elements *t* = (12, 20, ∞, ∞, ∞, …) weeks and *y* = (54, 164, ∇, ∇, ∇, …) millimetres.

There is a fundamental dependence between the marks *y* and the time-points *t* at which they are observed, for they arise together and neither can exist without the other. Dependence can also be associational and dynamic: writing *t̄_j_* for (*t*_1_, … , *t_j_*) and *ȳ_j_* for (*y*_1_, … , *y_j_*), a standard construction of marked point processes (Jacobsen, 2006, p. 22) specifies the conditional distributions (*t_j_* | *t̄*_*j*−1_, *ȳ*_*j*−1_) and (*y_j_* | *t̄_j_*, *ȳ*_*j*−1_) sequentially for *j* ⩾ 1.

**Fig. 1:**
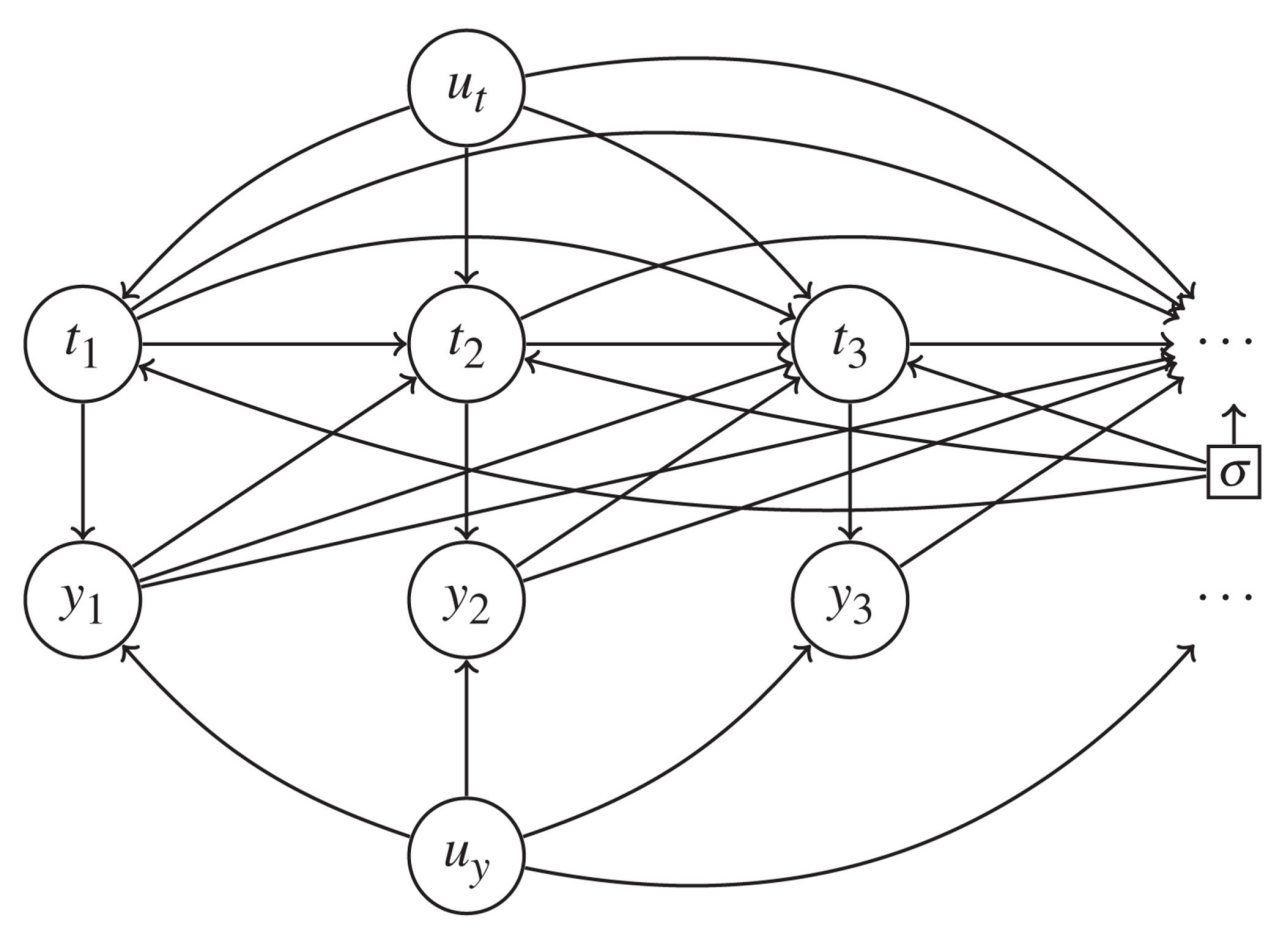
Influence diagram for Example 1: stable, because the observation time *t_j_* depends on previous times *t*_*j*−1_ and marks *ȳ*_*j*−1_, but not the unobserved *u_y_* that influence *y*.

We use directed acyclic graphs to summarize possible dynamic dependencies between *t* and *y*. Pearl (2009) demonstrates how such graphs may be given a causal interpretation; in addition to conveniently summarizing conditional independence statements that apply across a system of random variables, causal graphs also encode rules for transforming the probability measure describing the observed data into different measures that would apply under specific external interventions. Here, we use the related concept of influence diagrams (Dawid & Didelez, 2010). Influence diagrams contain a special node, *σ*, a parameter that governs the behaviour of the system. This *σ* indicates if the system is operating in its original, observed state (*σ* = *o*) or, when *σ* takes different values, whether particular interventions into the system are being considered. Under *σ* = *o*, probability functions are written pr(·; *σ* = *o*) or just pr(·). In general, arrows from *σ* into other nodes indicate that their stochastic behaviour may be altered under different regimes. We consider the specific case where arrows emanate from *σ* to every *t_j_*, and only to the *t_j_*. For a specific example, see Fig. 1. Our *σ* is equivalent to the *F* used in the appendix of Pearl (1995).

Our influence diagrams will contain nodes representing *t_j_* and *y_j_* for every *j*. Other nodes will usually appear; we shall write observed baseline covariates generically as *x*, with *u* denoting random variables whose realized values are not observed. Whatever the larger picture of dependence between *u*, *x*, *t* and *y*, the influence diagram should contain, for every *j*, the subgraphs *t_j_* → *y_j_*, in which a directed arrow from *t_j_* to *y_j_* formalizes the notion that *y_j_* comes into existence at time *t_j_* and that if *t_j_* is changed, so too is *y_j_*.

## 3 Ignorability

If dependence between *t* and *y* is indeed inherent in a marked point process, then it can never be dismissed entirely. Instead, we may ask when this dependence can safely be ignored. A popular approach to the analysis of longitudinal data (see, for example, Diggle et al., 2002) is based on generic multivariate models. To this end, and whether or not this is actually the case, it is customary to imagine that the occasions *t* on which we observe the sequence of measurements *y* are fixed by design (Molenberghs & Verbeke, 2006, p. 482). In the language of causal graphs and using the notation of Pearl (2009), this amounts to an interventionist view: instead of allowing *t* to evolve stochastically, we have intervened to enforce a particular pattern of observations do(*t*_1_), do(*t*_2_), … , or simply do(*t*). Circumstances in which the presence, *σ* = do(*t*), or absence, *σ* = *o*, of such intervention is irrelevant to inference might be deemed ignorable, and we make this our definition.

DEFINITION 1. *The point process t is ignorable for inference based on y if* pr(*t*, *y*) ∝ pr{*y*; do(*t*)} *with respect to some parameter of interest. More generally, the point process t is ignorable for inference based on y, conditional on x, if* pr(*t*, *y* | *x*) ∝ pr{*y* | *x*; do(*t*)} *with respect to some parameter of interest*.

If the two objects are indeed proportional, then they may be used interchangeably for likelihood-based inference. Establishing general conditions under which this proportionality holds is precisely the question addressed by Rubin (1976). To see this, consider Rubin’s statement (Rubin, 1976, p. 584):

Ignoring the process that causes missing data means proceeding by: (a) fixing the [missing data indicator] at the observed pattern of missing data […], and (b) assuming that the values of the observed data […] arose from the marginal density of the [observed data].

This refers to a derived, interventional, distribution. We interpret Rubin’s statement as an intent to employ pr{*y*; *σ* = do(*t*)} as the likelihood upon which inference is to be based. An immediate advantage of the causal formulation is that it provides the informative label pr{*y*; *σ* = do(*t*)}, or more simply pr{*y*; do(*t*)}, for the nameless integral construction that Rubin (1976) calls a marginal density. While pr{*y*; do(*t*)} is indeed a marginal probability function, it is computed with respect to the interventional regime *σ* = do(*t*) and is quite distinct from both the conditional pr(*y* | *t*) = pr(*y* | *t*; *σ* = *o*) and the more complicated marginal pr(*y*) = pr (*y*; *σ* = *o*).

Rubin answers the question of when proportionality between pr(*t*, *y*) and pr{*y*; do(*t*)} may be inferred through his condition known as missingness at random. However, because within a marked point process formulation there are no missing data, we require an analogous condition that does not employ notions of complete, observed and missing data. Like missingness at random, our contender, stability, is a simple, general, sufficient condition under which ignorability may be shown to hold. However, also like missingness at random, stability is not a necessary condition for ignorability; we return to this point in our discussion.

DEFINITION 2 (Dawid & Didelez, 2010). *The marked point process* (*t*, *y*) *exhibits simple stability if y_j_* ⫫ *σ* | (*x*, *t̄_j_*, *ȳ*_*j*−1_).

Here ⫫ denotes independence. While simple stability can be verified on an influence diagram by checking the relevant graphical separation, the fact that *σ* only has directed edges into the *t_j_* allows the following equivalent graphical check where *σ* is omitted from the graph: the marked point process is stable if there are no unblocked back-door paths between *t_j_* and *y_j_*, i.e., paths between *t_j_* and *y_j_*, with an arrow into *t_j_*, that are not blocked by any of (*x*, *t̄*_*j*−1_, *ȳ*_*j*−1_) in the sense of d-separation (Pearl, 2009).

THEOREM 1. *If the marked point process* (*t*, *y*) *is stable, then*

(1) $$\text{pr}\left(t,y\;|\;x\right)=\left\{\prod_{j=1}^{\infty }\text{pr}\left(t_{j}\;|\;x,{\bar{t}}_{j-1},{\bar{y}}_{j-1}\right)\right\}\times \text{pr}\left\{y\;|\;x;\text{do}(t)\right\}.$$

COROLLARY 1. *If the marked point process* (*t*, *y*) *is stable, then maximizations over parameters occurring in only one of its two likelihood factors may equivalently be performed over either the full likelihood* pr(*t*, *y* | *x*) or the relevant factor. In particular, any parameters that occur only in pr{*y* | *x*; do(*t*)} *may be maximized over this simpler likelihood.*

*Proof*. The standard decomposition for the likelihood of a marked point process is

$$\text{pr}\left(t,y\;|\;x\right)=\prod_{j=1}^{\infty }\text{pr}\left(t_{j}\;|\;x,{\bar{t}}_{j-1},{\bar{y}}_{j-1}\right)\;\text{pr}\left(y_{j}\;|\;x,{\bar{t}}_{j},{\bar{y}}_{j-1}\right).$$

Since (*t*, *y*) is stable, pr(*y_j_* | *x*, *t̄_j_*, *ȳ*_*j*−1_; *σ* = *o*) = pr{*y_j_* | *x*, *t̄_j_*, *ȳ*_*j*−1_; *σ* = do(*t*)}, whence

$$\text{pr}\left(t,y\;|\;x\right)=\prod_{j=1}^{\infty }\text{pr(}t_{j}\;|\;x,{\bar{t}}_{j-1},{\bar{y}}_{j-1}\text{)}\;\text{pr\{}y_{j}\;|\;x,{\bar{t}}_{j},{\bar{y}}_{j-1};\text{do}(t)\text{\}}.$$

But under *σ* = do(*t*), the *t_j_* are deterministic, so

$$\text{pr}\left(t,y\;|\;x\right)=\prod_{j=1}^{\infty }\text{pr(}t_{j}\;|\;x,{\bar{t}}_{j-1},{\bar{y}}_{j-1}\text{)}\;\text{pr\{}y_{j}\;|\;x,{\bar{y}}_{j-1};\text{do}(t)\text{\}}.$$

The infinite product ∏ pr{*y_j_* | *x*, *ȳ*_*j*−1_; do(*t*)} telescopes to yield the required factorization.

If the marked point process (*t*, *y*) is stable, likelihood inference may proceed solely on the basis of pr{*y* | *x*; do(*t*)}, provided that the parameters of ∏ pr(*t_j_* | *x*, *t̄*_*j*−1_, *ȳ*_*j*−1_) are suitably distinct from the parameters of interest in pr{*y* | *x*; do(*t*)}. It is possible, though unusual, that stability may plausibly be assumed to hold but the parameters of ∏ pr(*t_j_* | *x*, *t̄*_*j*−1_, *ȳ*_*j*−1_) and pr{*y* | *x*; do(*t*)} are not thought to be distinct. To see that this need not affect consistency of estimation, consider the following argument, due to Peter Diggle. Since parameterization is essentially a modelling decision, in this case we may consider the larger model in which any parameters in common are replaced by two variation-independent sets of parameters, one for each of the two likelihood factors. Providing parameters are replaced throughout any given conditional probability, the product of these conditional probabilities remains a probability measure that is consistent with the data-generating mechanism. It is then clear that estimation of the parameters of pr{*y* | *x*; do(*t*)} will still be consistent based only on maximizing over this component of the likelihood, albeit with some loss of efficiency relative to using the full likelihood pr(*t*, *y*).

The likelihood factorization of Theorem 1 is an example of G-computation (Robins, 1986). Indeed, rewriting (1) as

$$\text{pr}\left\{y\;|\;x;\text{do}(t)\right\}={\left\{\prod_{j=1}^{\infty }\text{pr}(t_{j}\;|\;x,{\bar{t}}_{j-1},{\bar{y}}_{j-1})\right\}}^{-1}\text{pr}\left(t,y\;|\;x\right)$$

illustrates the close connection between ignorability and the identification of causal quantities such as pr{*y* | *x*; do(*t*)}, in particular with their identification via adjustment for previous observations through so-called inverse probability weighting. Philosophically, we find it preferable to frame problems of ignorability in terms of causal inference, without reference to counterfactual missing data. In the next section, we argue also for the practical importance of this approach, particularly in assessing the plausibility of assumptions required for ignorability.

## 4 Working Example Revisted

Like missingness at random, stability is not nonparametrically testable (Molenberghs et al., 2008); that is, stability cannot be assessed solely on the basis of an empirical joint likelihood function of observed random variables. However, this need not be the end of the story; Pearl (2009, p. 40) distinguishes sharply between statistical and causal concepts, and would classify stability as an essentially causal assumption precisely because it cannot be discerned from a joint distribution. To assess such a causal assumption requires formal consideration of at least two regimes, *σ* = *o* and *σ* = do(*t*), perhaps by way of a causal graph or an influence diagram. This gives expert judgement a formal place in analysis, and here we give some examples to illustrate how an expert, loosely defined, might go about assessing the plausibility of stability within our working example of foetal crown-rump length.

*Example* 1. Consider an antenatal clinic in which the next recommended ultrasound scan date *t_j_* is set on the basis of foetal length measurements *ȳ*_*j*−1_ from previous scans. For instance, pregnancies falling within reference ranges for foetal length might follow a standard scan schedule, while those showing unusually slow or rapid foetal growth might be invited to attend more frequently. There may also be privately scheduled, parent-initiated scans, perhaps because of underlying anxiety or a particular concern, summarized by *u_t_* in Fig. 1.

Any lack of adherence to these appointments is then assumed to arise from external factors that play no discernible role in determining the foetal length measurements, for example school holidays or local traffic conditions. In this way an observation time *t_j_* depends structurally on previous observations times *t̄*_*j*−1_, previous observations *ȳ*_*j*−1_ and underlying parental influences *u_t_* but, given these, is independent of other past factors or processes, and in particular of *u_y_*.

Covariance between scan measurements *y* may be incorporated in the usual way, by means of shared random effects *u_y_*. This can be thought of as capturing the underlying physical characteristics of the unborn child; but, crucially, these underlying characteristics are only thought to influence scan dates indirectly, by way of their influence on the measurements *y*.

This is our canonical example of stability. Because *u_t_* and *u_y_* are marginally independent, it is straightforward to show from first principles that pr(*t*, *y*) equals

$$\left\{\sum_{u_{t}}\text{pr}\left(u_{t}\right)\prod_{j=1}^{\infty }\text{pr}\left(t_{j}\;|\;u_{t},t_{1},\ldots ,t_{j-1},y_{1},\ldots ,y_{j-1}\right)\right\}\times \;\left\{\sum_{u_{y}}\text{pr}\left(u_{y}\right)\prod_{j=1}^{\infty }\text{pr}\left(y_{j}\;|\;u_{y},t_{j}\right)\right\}.$$

The latter factor is precisely pr{*y*; do(*t*)}, and its form is the usual mixture distribution arising when random effects are integrated out of a likelihood function, as in Laird & Ware (1982) for example. Stability would be lost if *u_y_* had an arrow directly into any *t*_*j*_.

**Fig. 2:**
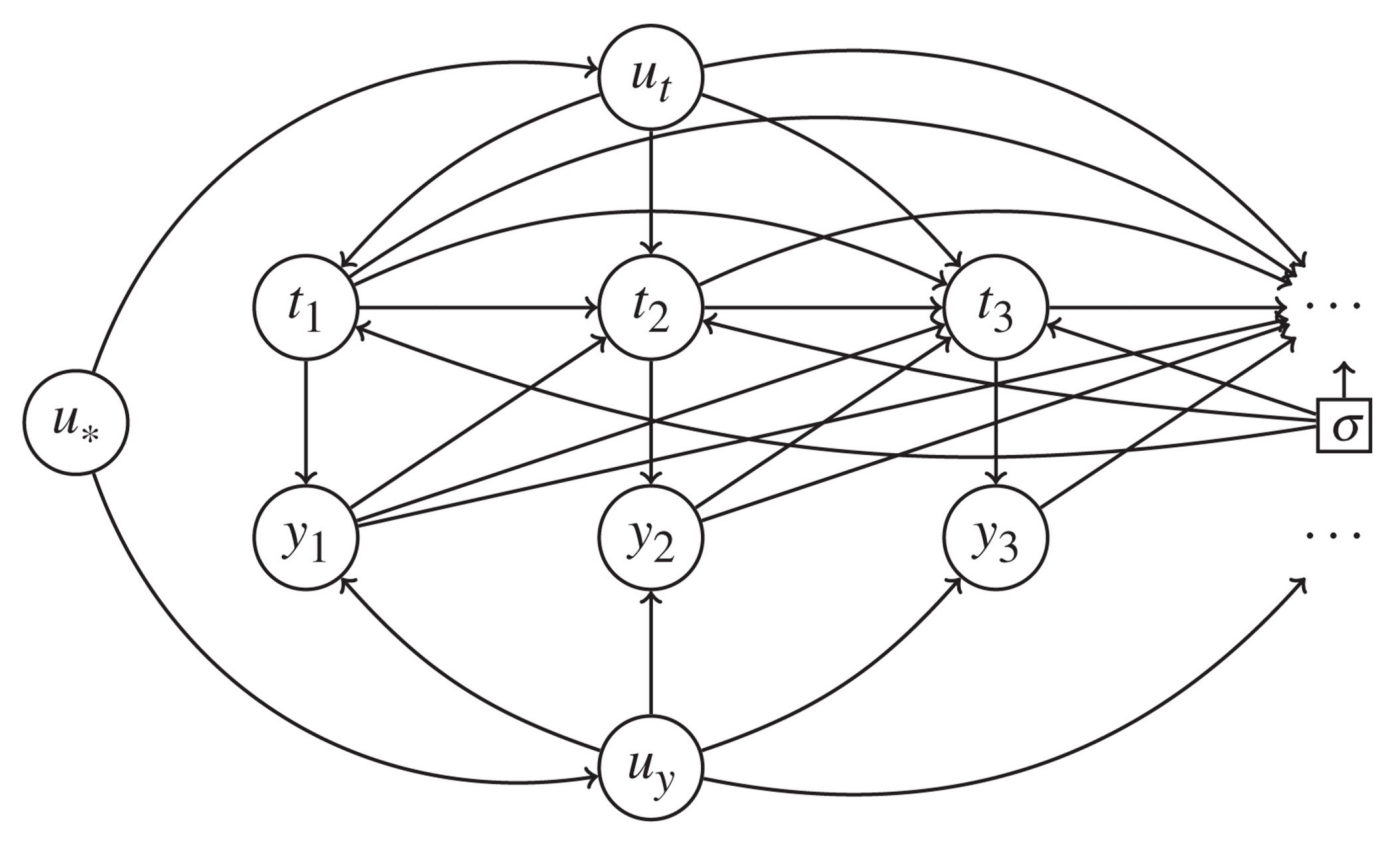
Influence diagram for Example 2: not stable, because *t* and *y* have correlated, unobserved parents *u_t_* and *u_y_*.

*Example* 2. The assumption that parental influences on the timings of antenatal measurements are independent of their unborn child’s growth is arguably a strong one. In particular, it seems at least plausible that a mother’s health, u_*_ say, could play some role in determining both her levels of antenatal anxiety *u_t_* and the growth of her child *u_y_*; such dependence is depicted in Fig. 2.

The marked point process (*t*, *y*) is now no longer stable: the likelihood function takes the form

$$\begin{array}{l} \sum_{u_{*}}\{\sum_{u_{t}}\text{pr}\left(u_{t}\;|\;u_{*}\right)\;\prod_{j=1}^{\infty }\text{pr}\left(t_{j}\;|\;u_{t},t_{1},\ldots ,t_{j-1},y_{1},\ldots ,y_{j-1}\right)\\ \;\times \sum_{u_{y}}\text{pr}\left(u_{y}\;|\;u_{*}\right)\prod_{j=1}^{\infty }\text{pr}\left(y_{j}\;|\;u_{y},t_{j}\right)\}, \end{array}$$

and no reduction to pr{*y*; do(*t*)} is possible. The timings *t* are not ignorable for inference about *y*, and inferences based on pr{*y*; do(*t*)} will in general be biased. Stability may be re-established if *u_t_* can be replaced by a set of measurements *x* assessing antenatal anxiety, assuming for the sake of argument that this can be done without appreciable error in measurement. Conditioning on this *x* breaks the dependency between *t* and *y*, and allows us to write pr(*t*, *y* | *x*) as

$$\prod_{j=1}^{\infty }\text{pr}\left(t_{j}\;|\;x,t_{1},\ldots ,t_{j-1},y_{1},\ldots ,y_{j-1}\right)\times \left\{\sum_{u_{*}}\text{pr}\left(u_{*}\;|\;x\right)\sum_{u_{y}}\text{pr}\left(u_{y}\;|\;u_{*}\right)\prod_{j=1}^{\infty }\text{pr}\left(y_{j}\;|\;u_{y},t_{j}\right)\right\}.$$

An important difference from the factorization in Example 1 is the conditioning on *x* in the mixing distribution pr(u_*_ | *x*). The second factor does, of course, reduce to pr{*y* | *x*; do(*t*)}.

**Fig. 3:**
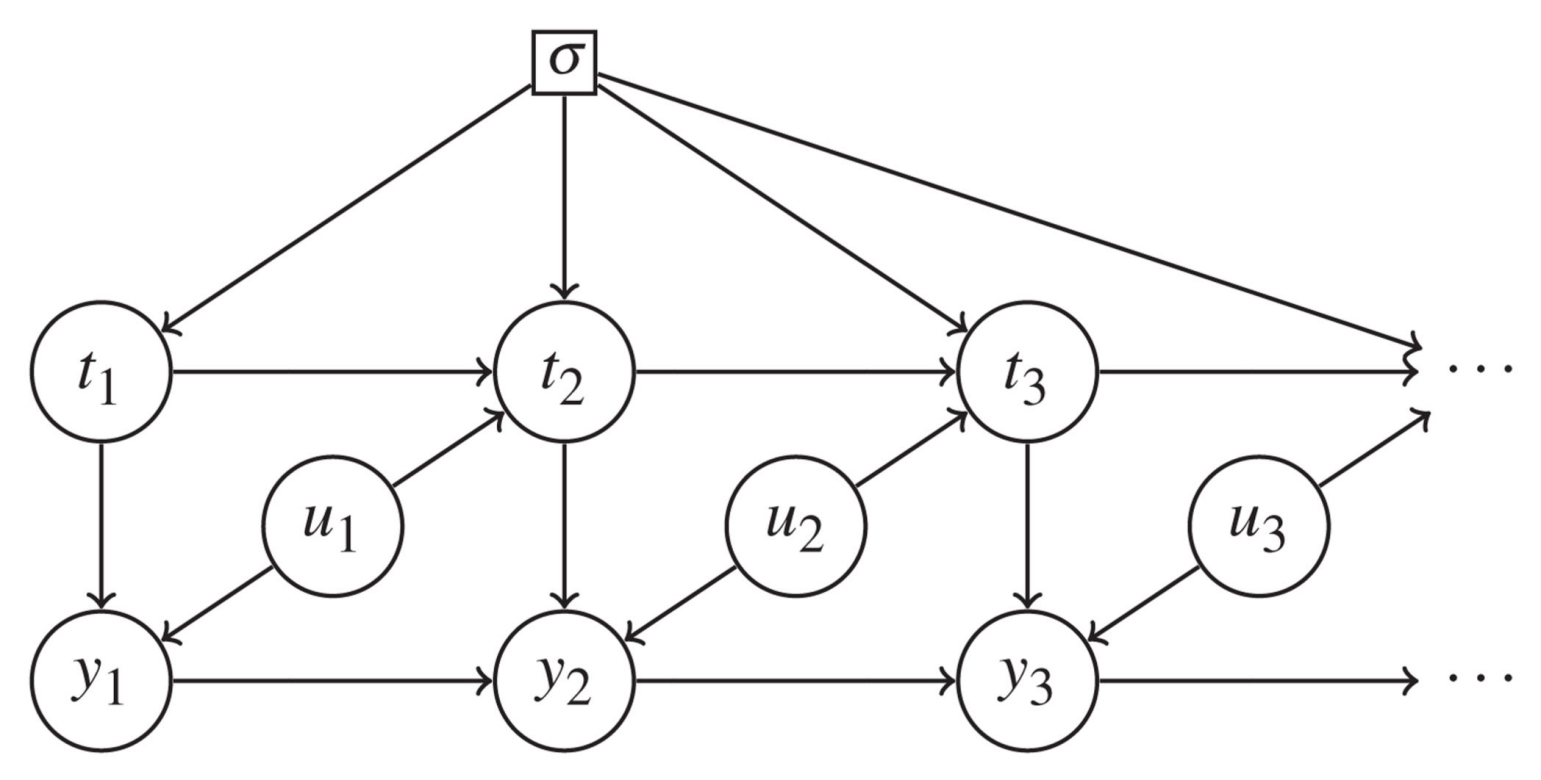
Influence diagram for Example 3: stable, because the unobserved *u*_*j*−1_ influences *y*_*j*−1_ and *t_j_* but not *y_j_*.

*Example* 3. Our final scenario gives rise to a more surprising example of stability. Suppose that clinic visits are scheduled for 12 and 20 weeks’ gestation, but if, at such a visit, the sonographer perceives the ultrasound equipment to be behaving unreliably, an additional measurement is arranged for the following week. No record is kept of equipment failure, so these enter the influence diagram as unobserved *u_j_*, as in Fig. 3. Failures are assumed to occur independently of any previous failures and of all other aspects of the system. Since equipment failure might perhaps make crown-rump length measurements larger, say, or more variable, *u_j_* influences *y_j_* in addition to affecting the subsequent *t*_*j*+1_.

Despite unobserved common causes between *t* and *y*, the marked point process (*t*, *y*) remains stable because the unobserved factor *u_j_* affecting *y_j_* influences only the future *t*_*j*+1_, not the current *t_j_*. Stability is immediately lost if equipment failures are not independent of one another.

Also important for stability in this case is the assumed autoregressive dependence structure of the *y_j_*. Stability would also be lost if this were replaced by the random effects *u_y_* of the previous two examples, a scenario that we find much more plausible.

## 5 Discussion

We contend that missing data are not nearly so widespread as their prominence in the statistical literature would imply. It is, of course, sensible to formulate stochastic systems in terms of unobserved random variables. However, describing such unobserved variables as data seems to us appropriate only when they were at some time, by some means, given a specific value that could in principle have been observed, even if that value has subsequently become lost to us. This perspective can be traced back to the dawn of the missing data literature, as Rubin (2014, p. 598) recollects:

[David Cox, then editor of *Biometrika*] mentioned that he really wasn’t fond of the title of the already accepted Rubin (1976) because something that’s missing can’t be “given” – the Latin meaning of data.

It seems to us that the missing data label might reasonably be applied if data were actually gathered but subsequently lost. An obvious example would be clinical data lost when a laptop computer disappears from a crowded train. In this rather specific sense, missing data do sometimes exist.

Formulations of ignorability that rely on missing data require the user to assign meaning to these quantities in order that the plausibility of assumptions concerning the missing data can be assessed. Little and Rubin observed that the ability to assign such meaning formally underpins the majority of their influential book (Little & Rubin, 2002, p. 8, Assumption 1.1) and most related work: ‘missing indicators hide true values that are meaningful for analysis’. We believe that removing reliance on assigning meaning to missing data makes our assumptions easier to understand and evaluate.

Often missing data are given a counterfactual interpretation, especially in the longitudinal setting. It is widespread practice to employ a notional variable *y_j_* recording the value that would have been observed had a measurement taken place at time *t_j_*. In some instances it may be possible to make such notions concrete, particularly if errors in measurement are negligible. However, such a formulation requires some understanding of why these hypothetical measurements took place: did the subject become sufficiently well, or unwell, to allow or require measurement? How ought we to allow for the multiplicity of reasons for which a measurement might have taken place but in fact did not occur? In order to assess a condition such as missingness at random, dependence of the missingness mechanism on these infinite-dimensional, vaguely defined counterfactuals must be examined, which is arguably a daunting task.

Shpitser et al. (2015) also argued for causal reasoning about questions of ignorability, and the forthcoming book by Hernan & Robins (2017) employs inverse probability weighting to address closely related problems. Both theories, though, are based on counterfactual missing data. Our view is that inverse probability weighting becomes even more natural when weighting is done not in order to restore fictitious missing data, but by the probability that the observed data arise as they do.

Our unobserved nodes *u* play an important role in assessing stability. We might variously choose to think of these as infinite-dimensional objects summarizing the entire trajectory of an unobserved, possibly highly multivariate, stochastic process, or alternatively as very low-dimensional objects, for instance a random intercept and slope. The former perspective is useful in assessing assumptions, while the latter is more suited to applied statistical modelling. Dawid & Didelez (2010) extended the notion of simple stability to a similar, but weaker, assumption that involves conditioning on such unobserved nodes. This weaker version may then be combined with other assumptions to regain simple stability, while in other cases simple stability fails but adjustment is still possible, and hence proportionality of likelihoods may still be shown to hold. We reiterate that, as with missingness at random, stability is not necessary for ignorability.

Dawid & Didelez (2010) also admit what might be called time-varying covariates. This extension is possible here, too, but would require that similar consideration be given to the occasions and reasons that such covariates were measured.

We have argued that it is useful to think of longitudinal data in terms of marked point processes, especially when there may be dependence between points and marks. Even in the absence of such dependence, it seems to us quite natural to base inference within a stochastic process setting. Most fundamentally, time is given a central role (Aalen, 2012), which is especially important in causal reasoning. This is in contrast to the usual multivariate modelling of longitudinal data, where although time may be given a conspicuous notational presence, its inferential role is often restricted to forming suitable covariance structures. Other advantages of marked point process models for longitudinal data include elegant martingale decompositions analogous to those in widespread use in event-history analysis (Martinussen & Scheike, 2007).

In the context of longitudinal data, missingness at random has been defined in various ways and with varying degrees of formality. Many authors employ informal notation such as *y*_obs_ and *y*_mis_ to refer to observed and unobserved components, and data are said to be missing at random if pr(*t* | *y*_obs_, *y*_mis_) = pr(*t* | *y*_obs_). Since we have avoided defining complete data, we could not use this notation here. This is no bad thing, as the *y*_obs_, *y*_mis_ notation is at best ambiguous and at worst confusing (Seaman et al., 2013; Mealli & Rubin, 2015); taken literally, *y*_obs_ must at least encode the value of *m* and, in balanced monotone drop-out cases, actually determines *t* completely.

Many variants of missingness at random, such as covariate-dependent missingness at random (Little, 1995) or sequential missingness at random (Robins et al., 1995; Hogan et al., 2004), are subsumed within the general approach outlined here. Although we have focused on longitudinal data, the formulation of ignorability given in the present paper also applies in other settings: for instance, spatial point processes may raise similar questions of informative sampling. Censoring, and more generally coarsening (Heitjan, 1994), could also be formulated in these terms. We emphasize again the advantage of making explicit the observational and interventional likelihoods whose proportionality is in question.
